# Study of iron metabolism based on T2* mapping sequences in PI-RADS 3 prostate lesions

**DOI:** 10.3389/fonc.2023.1185057

**Published:** 2023-05-18

**Authors:** Wenhao D, Guangzheng L, Zhen T, Xuedong W, Yonggang L, Xuefeng Z, Weijie Z, Gang L, Yuhua H

**Affiliations:** ^1^ Department of Urology, The First Affiliated Hospital of Soochow University, Suzhou, China; ^2^ Department of Radiology, The First Affiliated Hospital of Soochow University, Suzhou, China

**Keywords:** prostate cancer, PI-RADS, iron mentalism, multiparametric magnetic resonance imaging, diagnosis

## Abstract

**Introduction:**

Prostate cancer is one of the most common malignant tumors in Chinese men, which is rich in iron metabolic activity and is closely related to all stages of prostate cancer progression. Since the current diagnostic methods are insufficient, we aimed to evaluate the value of quantitative T2 star values from the T2* mapping sequences in multiparametric magnetic resonance imaging (mpMRI) in the diagnosis and grading of PI-RADS 3 prostate cancer (PCa).

**Methods:**

We prospectively enrolled patients with PCa or benign prostatic hyperplasia (BPH) admitted to our hospital from January 2021 to November 2022. Imaging indicators, including the T2* value and apparent diffusion coefficient (ADC) value, were collected, and enzyme-linked immunosorbent assays (ELISAs) were used to measure the levels of proteins involved in iron metabolism in the patients. ROC curves were drawn to explore whether the T2* value could be used for the diagnosis and grading of PCa.

**Results:**

We found that three iron metabolism indexes, ferritin, hepcidin, and the ferric ion (Fe), and the T2* value were significantly different between the PCa group and BPH group and between the low International Society of Urology Pathology (ISUP) group (ISUP ≤ 2) and the high ISUP group (ISUP>2). Additionally, there was a significant correlation between the levels of these three indicators and the T2* value. Further ROC analysis showed that the levels of iron metabolism-related indexes and T2* values performed well in diagnosing and grading PCa.

**Discussion:**

The T2* value has good value in detecting and predicting the grade of prostate cancer and can reflect the iron metabolism of the tumor, which could provide a foundation for the diagnosis and grading of PCa in the future.

## 1 Introduction

Prostate cancer (PCa) is one of the most common malignancies in male and the second leading cause of cancer-related death in adult men worldwide; in China, PCa ranks ninth in the incidence of male malignancies (1). According to CA-A CANCER JOURNAL FOR CLINICIANS ‘s forecast, there will be 288,300 new cases of PCa in the United States in 2023, and 34,700 patients will die of prostate cancer2200 (2). Nearly 30% of new cancer cases are PCa. More importantly, despite the continuous progress of prostate diagnostic technology, the incidence of PCa in China is increasing yearly, and the proportion of advanced prostate cancer is significantly higher than that in other countries. This may be due to the limitations of screening and the high rate of missed diagnosis. Many patients are elderly, and because of the subtle nature of PCa symptoms, they are diagnosed for the first time because of frequent urination and other lower urinary tract symptoms (LUTS). Therefore, the accurate screening of PCa in patients with benign prostatic hyperplasia is the most important method to improve the detection rate of PCa.

To date, the diagnosis of PCa mainly depends on two methods: the measurement of serum total prostate-specific antigen (TPSA) levels and multiparametric magnetic resonance imaging (mpMRI). In recent years, noninvasive methods, such as PSA measurements, have been developed for evaluating preoperative PCa lesions, and the diagnostic value of these methods for determining progression and prognosis has been evaluated. Although TPSA assays have high sensitivity, their low specificity has led to the overuse of prostate biopsy. Therefore, improving the efficiency of PCa diagnosis and avoiding unnecessary invasive examinations are pivotal components of the diagnosis and treatment of PCa. mpMRI of the prostate is currently another important component of noninvasive PCa diagnosis. mpMRI is not burdened by the economic costs associated with the surgical injury caused by prostate biopsy or false-positive results and can be comprehensively performed before surgery to assess the location, boundaries, and environment of the tumor. The apparent diffusion coefficient (ADC) value has also been discussed with regard to its relation to the pathological stage and prognosis of PCa (3). PI-RADS is currently one of the most widely used scoring criteria for PCa. It is used to evaluate the likelihood of focal prostate nodules to be PCa by scoring T2 and diffusion-weighted imaging (DWI) sequences. According to the difference in high and low signals and clarity, the PI-RADS divides the prostate score into five grades, of which the third grade represents possible PCa. Although patients with third grade tumors exhibit clear qualitative criteria, there are subjective differences to a certain extent, the requirements for the center are higher, and different radiologists may have different opinions. Moreover, some PCa lesions with 2- or 4-point characteristics are often included in the 3-point category, in which there are a large number of incorrect scores. In addition, there is still a debate about whether lesions with PI-RADS3 scores need invasive puncture. According to studies, the positive rate of PCa puncture with PI-RADS3 patients is only 20%, which greatly affects the detection of PCa. Previous studies have assessed the prevalence of PCa in PI-RADS3 lesions and found that PI-RADS scores do not provide accurate guidance for clinical management (with or without biopsies), and the rate of missed diagnosis is the main problem at present. For patients with PI-RADS3, the ambiguous PI-RADS score does not represent a better prognosis than the higher PI-RADS grade. Although the ADC value can help to judge the malignancy of malignant prostate tumors to some extent, it has obvious limitations. Some PI-RADS3 patients often have a very poor International Society of Urology Pathology (ISUP) grade, which seriously affects their survival and prognosis. Therefore, the two existing noninvasive examination items cannot provide effective guidance on the PI-RADS score, and the diagnosis of PI-RADS3 score of PCa is still in the exploratory stage (4–6). So far, many tools, such as biomarkers, associated with mpMRI have aimed to solved this particular problem, such as SelectMDx (7), 4Kscore, ExosomeDx™ (8) and PCA3 (9). These biomarkers can improve the specificity of PCa by combining with mpMRI, and have a significant improvement compared with traditional TPSA or prostate-specific antigen density (PSAD).

With the in-depth study of iron metabolism, increasing evidence has shown a correlation between iron metabolism and the occurrence and progression of malignant tumors (10). Iron is one of the basic nutrients needed by cancer cells. When tumor cells are in an iron-rich environment, the growth and invasion of cancer cells are significantly faster than those in an iron-deficient environment. However, too much iron can cause another problem: oxidative damage to cancer cells. When cancer cells are exposed to too much iron, iron promotes another phenomenon by activating oxidative damage: iron death, a mechanism that damages the structure of cancer cells. However, cancer cells form protective mechanisms against oxidative damage and iron death, which are different in all types of cancers and have similar mechanisms in PCa. In cancer cells, the activity of antioxidant enzymes is increased, so cancer cells do not immediately undergo the killing caused by fast-acting iron in the iron-rich environment, so a very large amount of iron is needed to cause the death of cancer cells. Cancer cells use iron for important biochemical reactions, such as DNA synthesis, mitochondrial metabolism, angiogenesis and metastatic cell proliferation. In PCa, iron is also very important for the occurrence and development of tumors. Like other tumors, the growth of PCa cells requires sufficient iron, which can activate enzymes that control the transcriptional activity of androgen receptor (AR) in PCa, which is an important initiating factor. Moreover, iron can reactivate the activity of enzymes in cancer cells, thereby increasing energy production and extracellular matrix degradation. Recent studies have confirmed that the content of iron in PCa cells is increased, while in normal cells near PCa cells, iron levels are lower (11). Many kinds of iron metabolism molecules have been shown to promote or inhibit the progression of PCa. For patients with PI-RADS3 prostate disease whose imaging results are unclear, whether the difference in iron metabolism can help to improve the detection rate of PI-RADS3 is a direction that needs attention to guide clinical diagnosis and treatment from a microscopic point of view.

Here, we introduce a less-used MR sequence in urology, since the unclear anatomical division, difficulty in parameter adjustment, small prostate volume and so on. The T2* mapping sequence was initially used to assess iron deposition in the heart and spleen (12). In the context of liver surgery, the T2* mapping sequence can be used to quantitatively determine liver iron deposition and iron overload based on the difference in T2 relaxation time and has better accuracy than liver biopsy (13). MRI signal decay is affected by the iron content of the tissue; the higher the iron content, the faster the signal decay. In turn, the T2* value represents the iron content as represented by the R2* relaxation rate (14). In the field of PCa, whether the T2* mapping sequence can increase the detection rate of PCa from a new perspective by predicting iron metabolism in patients with PI-RADS3 is unknown. In summary, the assessment of PI-RADS3 seems to have become a key challenge, and a large number of patients with PCa that cannot be diagnosed by TPSA or mpMRI based on T2+DWI sequences are included in this category. This limitation greatly affects the detection rate and prognosis of these patients, and a new method needs to be introduced to address this problem. In the field of urology, whether T2* mapping can be used to evaluate prostatic iron deposition to help diagnose prostate malignant tumors and even evaluate prognosis remains unknown. Therefore, the aim of this study was to prospectively evaluate the role of quantitative measurement of intratumoral iron deposition based on T2* mapping sequence as a noninvasive biomarker of iron metabolism in PCa with PI-RADS 3.

## 2 Materials and methods

### 2.1 Participants

**Figure 1:**
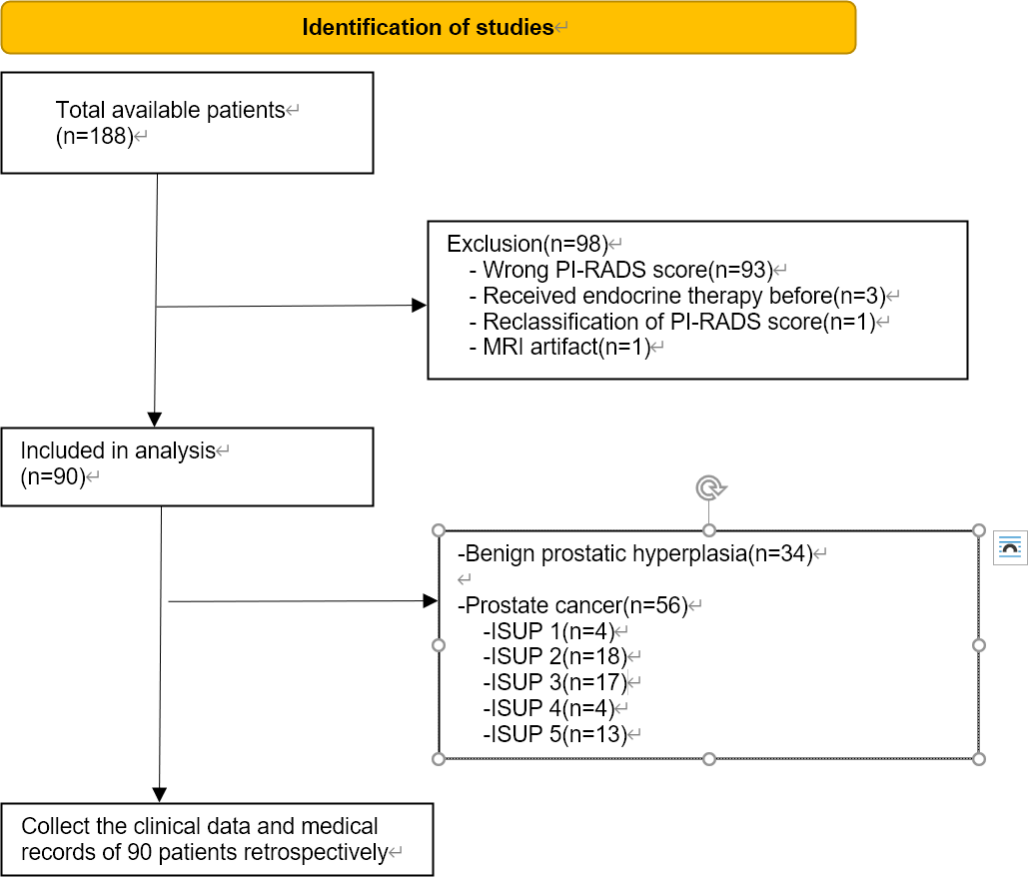
PRISMA flow diagram of patients involved and order of the processes.

This prospective study was approved by the Medical Ethics Committee of the First Affiliated Hospital of Soochow University (Suzhou, China; 2021; No. 133). Written informed consent was obtained from all the patients. Patients were included from January 2021 to November 2022. Patients hospitalized in the First Affiliated Hospital of Soochow University diagnosed with prostate diseases were prospectively subjected to mpMRI before prostate biopsy. The sequence included T2, DWI, ADC and T2* mapping. Serum samples were also collected. Two radiologists rescored all patients based on PI-RADS and included patients with PI-RADS3 in this study. Then, retrospective collection of data regarding the clinical indicators of patients in this study, including TPSA levels, prostate volume, pathological grade and others, was performed. The inclusion criteria were as follows: (1) MRI of the prostate, including T2-weighted imaging (T2WI), DWI, and T2* mapping-weighted imaging and surgery- (laparoscopic radical prostatectomy or transurethral resection of prostate) and postoperative pathology-confirmed PI-RADS 3 PCa or benign prostatic hyperplasia (BPH); (2) MRI examination at our hospital less than six weeks after prostate surgery; and (3) The lack of acute hepatitis or blood diseases affecting iron metabolism. Exclusion criteria were as follows: (1) treatment for PCa before surgery, such as endocrine therapy or radiotherapy; (2) other diseases affecting iron metabolism except PCa; and (3) film reading hampered by MRI artifacts. Based on the primary criteria, our study included 90 patients, including 56 with PCa and 34 with BPH. Ninety-three patients were excluded because of their incorrect PI-RADS score, three patients were excluded because they had received endocrine therapy, one patient was excluded due to an MRI artifact, and one patient was excluded due to reclassification of the PI-RADS score. In accordance with the PCa grading system, patients were divided into five categories. Grades assigned by the ISUP to patients 1, 2, 3, 4, and 5 were 4, 18, 17, 4, and 13, respectively. The PRISMA flow chart was shown in **Figure 1**.

### 2.2 Serum and tissue samples

Preoperative blood was collected from the two groups of patients. Serum and erythrocytes were rapidly and carefully separated by centrifugation at 3000 rpm for 10 minutes. The expression levels of ferritin and hepcidin in serum were measured by enzyme-linked immunosorbent assay (ELISA). After the prostate tissue was acquired after laparoscopic radical prostatectomy or transurethral resection of prostate, the specimens were then mashed with an appropriate amount of normal saline. The supernatant was collected by centrifugation at 3000 rpm for 10 minutes, and the tissue homogenate was detected by ELISA. The process was carried out in strict accordance with the kit instructions.

### 2.3 MRI protocol

Examinations were performed by using a 3.0 T clinical MR scanner (Skyra; Siemens Medical, Germany) with a dedicated 16-channel body-phased array coil. All images of 90 patients were assessed by 2 physicians respectively based on the PI-RADS score. Through the former study and following practice (15), an axial fast spin echo T2-weighted sequence was performed with the following parameters: repetition time/echo time (TR/TE) 7590/104 ms, slice number 25, slice thickness 3 mm, intersection gap 0 mm, field of view (FOV) 200 mm, voxel size 0.5*0.5*3 and flip angle 120°. T2* relaxation time maps were obtained using a multiecho fast field sequence. The parameters used were as follows: TR 265 ms, TE 4.92, 7.38, 9.84, 12.30, and 14.76 ms, slice number 30, slice thickness 3 mm, intersection gap 0.6 mm, FOV 380 mm, voxel size 1.5*1.5*3 and flip angle 50°.

### 2.4 PI-RADS score

The images obtained from the mpMRI scans were transferred to the Picture Archiving and Communication System (PACS), and the scores were rescored according to the PI-RADS V2.0 by 2 physicians with 10 years of experience in prostate MR diagnosis. The raters knew the patient’s baseline data but were blinded to the pathology results.

### 2.5 Correlation of the T2* value

The slice that showed the greatest extent of the lesion area was selected on the PACS, and the region of interest (ROI) was set. For multiple suspected tumor sites, the one with lowest T2* value was eventually selected for delineating the ROI. Considering the difficulty of sampling, we generally select an area of 1cm*1cm-sized circular area as the ROI. For tumors with too large lesions, we use the area with the lowest T2* value. Each measurement was repeated 3 times, and the average value was taken. After prostatectomy, each prostate pathology image was divided into 5 mm thick slices. Using the corresponding positions on the MR images, experienced pathologists manually marked six points on the prostate pathology images—the basal section, the apex, the peripheral zone, the central gland, the tip and the urinary tract—which were aligned with the corresponding parts on the MRI images. Symbols were used to identify various distinct morphological characteristics and then used to align the images with the step-section slices. For patients with BPH, the prostate was also corresponded to the six positions as described above, and the average value of the 1cm*1cm circular area of ​​the transitional zone of hyperplasia is taken.

### 2.6 Data analyses

All data were tested with SPSS 22.0 software (IBM, Armonk, NY, USA). According to the normality test, the baseline data do not fit a normal distribution, and are presented as medians (interquartile range), and group comparisons were made using nonparametric tests. Spearman correlation was used for correlation analysis. Taking the pathological results as the gold standard, the ROC curve was drawn. The difference was considered statistically significant at p<0.05, and applied to all evaluations.

## 3 Results

### 3.1 Patient characteristics

The pathological findings of 90 patients were included in this prospective study, including 34 patients with BPH and 56 patients with PCa. To explore differences in patient clinical data, we compared the TPSA, ratio of free to total PSA (F/TPSA), prostate volume, apparent diffusion coefficient (ADC) value, T2* value, ferritin, hepcidin, and Fe between the BPH and Pica groups. The results showed that the differences for all indicators between the two groups were statistically significant (all p<0.05; **Table 1**). The PCa group had higher TPSA, ferritin, hepcidin, and Fe levels than the BPH group, while the FTPSA, ADC value, T2* value, and prostate volume were lower than those of the BPH group.

**Table 1:** Patient clinical data [medians (interquartile range)].

Characteristic	Pca	BPH	P
Patient(n)	56	34	–
TPSA(ng ml^-1^)	15.85 (8.31,25.77)	8 (4.86,12.45)	<0.001
F/TPSA	0.12 (0.08,0.15)	0.15 (0.12,0.22)	0.019
Prostate volume(cm^3^)	34.85 (28.3,51.59)	59.08 (38.45,73.96)	0.001
ADC(*10^-3^ mm^2^/s)	0.695 (0.629,0.768)	0.756 (0.696,0.865)	0.02
T2*(ms)	42.02 (29.76,47)	54.34 (47.46,57.68)	<0.001
Ferritin(ng ml^-1^)	98.03 (82.92,113.69)	84.03 (78.05,92.49)	0.001
Hepcidin(ng ml^-1^)	114.12 (97.34,126.51)	100.74 (94.73,109.85)	0.008
Fe(μmol ml^-1^)	31.78 (27.27,36.07)	25.11 (22.52,30.03)	<0.001

TPSA, Total prostate-specific antigen; F/TPSA, Ratio of free to total PSA; ADC, Apparent diffusion coefficient; Fe, Ferric ion.

Considering that active monitoring (AS) can be selected for ISUP 1 and some ISUP 2 lesions, to avoid unnecessary repeated puncture and radical surgery, we further divided PCa patients into ISUP ≤ 2 and ISUP > 2 (**Table 2**). No statistically significant differences in TPSA or prostate volume were found between the two groups (p=0.09 and p=0.151, respectively). The rest of the indicators were significantly different.

**Table 2:** PCa Patient clinical data [medians (interquartile range)].

Characteristic	ISUP ≤ 2	ISUP>2	P
Patient(n)	22	34	–
tPSA(ng ml^-1^)	14.67 (6.58,22.42)	17.34 (11.37,32.87)	0.09
F/TPSA	0.14 (0.12,0.19)	0.1 (0.08,0.14)	0.017
Prostate volume(cm^3^)	43.95 (34.02,52.96)	32.41 (25.35,49.49)	0.151
ADC(*10^-3^ mm^2^/s)	0.760 (0.684,0.877)	0.671 (0.619,0.729)	0.004
T2*(ms)	47.03 (43.46,49)	34.57 (28.13,42.1)	<0.001
Ferritin(ng ml^-1^)	88.51 (73.13,98.56)	109.08 (93.5,116.87)	0.002
Hepcidin(ng ml^-1^)	105.73 (77.68,115.32)	117.16 (105.21,132.06)	0.012
Fe(μmol ml^-1^)	28.1 (23.56,32.05)	33.42 (29.51,37.53)	0.001
Nerve invasion(n)	14 (63.6%)	25 (73.5%)	0.889
Magin invasion(n)	8 (36.4%)	13 (38.2%)	0.436

TPSA, Total prostate-specific antigen; F/TPSA, Ratio of free to total PSA; ADC, Apparent diffusion coefficient; Fe, Ferric ion.

### 3.2 Correlation of BPH patient indicators with TPSA and T2* values

Next, we analyzed the correlation between the levels of serum markers and prostate volume and TPSA levels or T2* value, and the results showed that in BPH patients, the TPSA level was correlated with the prostate volume (p<0.001; **Table 3**), while no correlations were observed between the remaining markers and either TPSA or the T2* value (both P > 0.05).

**Table 3:** Associations between various parameters and TPSA or the T2* value in BPH.

Characteristic	TPSA		F/TPSA		Prostate volume		Ferritin		Hepcidin		Fe	
	r	p	r	p	r	p	r	p	r	p	r	p
T2* value	-0.222	0.206	0.159	0.243	-0.099	0.577	0.075	0.673	0.062	0.726	-0.33	0.057
TPSA	–	–	-0.202	0.134	0.695	<0.001	0.041	0.817	0.127	0.475	0.111	0.531

TPSA, Total prostate-specific antigen; ADC, Apparent diffusion coefficient; Fe, Ferric ion.

### 3.3 Correlation of PCa patient indicators with ISUP and T2* values

Next, we further analyzed the correlation between PCa patient indicators and ISUP grade and T2* value, and the results showed that TPSA, ADC value, ferritin, hepcidin, and Fe were all correlated with ISUP grade and T2* value. It is worth noting that the correlation coefficients of the T2* value, ferritin, hepcidin, and Fe with ISUP were -0.661, 0.52, 0.411, and 0.535, respectively (all p< 0.01; **Table 4**). Both the T2* mapping sequence and iron-related indexes better predicted the ISUP grade of PCa patients and performed well in evaluating the prognosis of patients. In addition, a correlation between the T2* value and ferritin, hepcidin, and Fe was observed (**Table 4**).

**Table 4:** Associations between indicators and ISUP grade or T2* value in PCa.

Characteristic	TPSA		T2* Value		ADC Value		Ferritin		Hepcidin		Fe	
	r	p	r	p	r	p	r	p	r	p	r	p
ISUP	0.349	0.008	-0.661	<0.001	-0.432	0.001	0.52	<0.001	0.411	0.002	0.535	<0.001
T2* Value	-0.386	0.003	–	–	0.482	<0.001	-0.441	0.001	-0.324	0.015	-0.541	<0.001

TPSA, Total prostate-specific antigen; ADC, Apparent diffusion coefficient; Fe, Ferric ion; ISUP, International Society of Urology Pathology.

### 3.4 T2* value in diagnosing PCa

**Figure 2:**
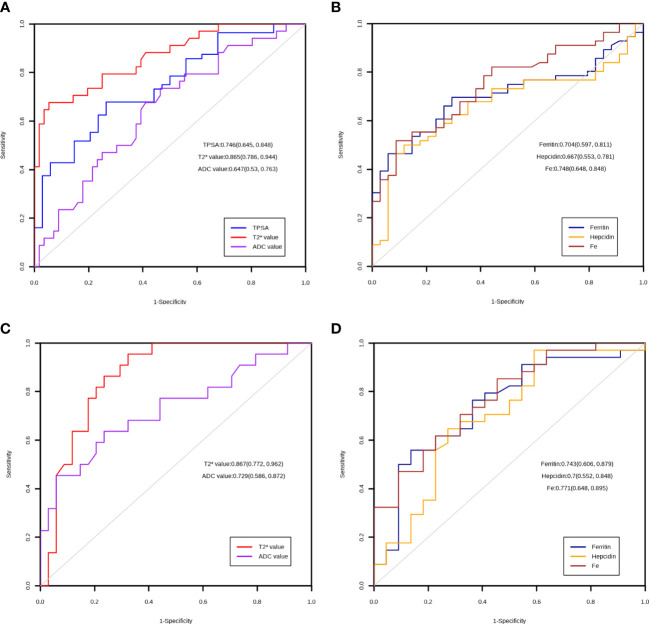
ROC curve of the markers. **(A)** Diagnostic utility of TPSA, T2* value and ADC value in the PCA and BPH group. **(B)** Diagnostic utility of ferritin, hepcidin and Fe in the PCA and BPH group. **(C)** Diagnostic utility of T2* value and ADC value in the low ISUP and high ISUP group. **(D)** Diagnostic utility of ferritin, hepcidin and Fe in the low ISUP and high ISUP group.

Then, we investigated the diagnostic value of the T2* value for PCa, as shown in **Figure 2A**. The ROC curve showed that the T2* value performed well in distinguishing PCa and BPH (AUC=0.865, p<0.001), while the TPSA, ADC value, ferritin, hepcidin, and Fe had AUCs of 0.746, 0.647, 0.704, 0.667, and 0.748, respectively (**Figure 2B**). This finding suggests that TPSA, ferritin and Fe also have good performance in the diagnosis of PCa.

### 3.5 The T2* value predicts ISUP grade in PCa patients

Finally, we explored the role of the T2* value in discriminating between PCa patients with ISUP ≤2 and >2. The ROC analysis results showed that the T2* value could significantly differentiate between the grades of PCa (AUC=0.867, p<0.001); the AUCs of the other markers are shown in **Figures 2C, D**.

## 4 Discussion

Prostate cancer morbidity and mortality are rising in Asia, with current diagnoses mainly relying on PSA, digital rectal exam (DRE) and mpMRI, as as reported above, posing a new challenge to PCa diagnosis. The focus of the diagnosis of PCa is to differentiate it from BPH, which is associated with symptoms that often mask the existence of PCa. With the advancement of MRI technology, mpMRI has become an effective modality for the noninvasive diagnosis of PCa. The existing routine sequence and the general qualitative PI-RADS scoring model are gradually becoming inadequate for meeting current requirements. Whether needle biopsy should be performed for a PI-RADS score of 3 and the positive rate of needle biopsy were not satisfactory. A 2016 multicenter prospective study showed that the detection rates of PCa were 13%, 31%, and 71% for lesions with a PI-RADS score greater than or equal to 3, respectively (16); other studies have made similar arguments (17–19). Interestingly, a study showed that for PI-RADS3 patients, dividing them into low-risk and high-risk groups according to the 0.5 ml threshold of lesion volume may help physicians make clinical decisions, but there is no large-scale central study to confirm this, and studies are mostly limited to the T1-2 clinical stage (20). Therefore, for PCa patients with PI-RADS 3, the lower detection rate of conventional MR sequences affects the diagnosis of early-stage PCa or clinically significant PCa. In the classic T2+DWI sequence, patients with PI-RADS 3 mostly showed heterogeneous low signals on T2 sequences and isointensity or mild hyperintensity on high b-value DWI sequences. In a previous study, the sensitivity of the T2 sequence + DWI sequence was significantly higher than that of the T2 sequence alone (81% vs. 54%, p<0.01), and the specificity of the T2 sequence + DWI sequence and T2 sequence alone was basically the same (21). DWI reveals obvious differences in the ADC values of patients with PCa and BPH, but there is a large overlap. Consequently, the ADC value alone is not recommended for the differential diagnosis of prostate diseases (22, 23). Models incorporating the qualitative ADC value and qualitative PI-RADS score have improved diagnostic efficiency over the PI-RADS score alone (24). At present, the detection rate of the conventional T2 sequence + DWI sequence is affected by the disease characteristics of the population, the quality of MR imaging, the experience of the reader, and the accuracy of prostate biopsy.

Angiogenesis and tumor metastasis are closely related to cellular iron metabolism. It has been proven that reducing intracellular iron metabolism inhibits tumor cell growth in both hormone-dependent and hormone-resistant cells (25). As a key factor in iron metabolism, hepcidin plays a crucial role, as it regulates the ferritin receptor on the cell membrane, preventing iron from leaving the cell. Subsequently, there is an increase in free iron production in tumor cells, which increases tumor cell invasiveness and promotes tumor cell growth. In this study, a higher level of serum hepcidin was found in the PCa group. In addition, the serum hepcidin levels were correlated with the ISUP grade. Elevated ferritin levels have been reported in other tumors (26–28), and in urine, ferritin heavy and light chains were confirmed to be different between the PCa and BPH groups. However, the effects of serum ferritin on PCa stage, progression, and prognosis need further experimental verification. In our study, the serum ferritin level was positively correlated with ISUP grade. Compared with systemic iron metabolism, local total Fe in prostate tissue can better reflect the significance of iron metabolism in PCa due to the presence of many confounding factors. At present, most studies have collected data on the trace iron in blood, and there are few studies on Fe in tissues. However, many experiments have confirmed that the iron content of PCa cells increases, and there is often iron overload (25). In this study, Fe level was positively correlated with ISUP grade and negatively correlated with T2* value; that is, the T2* value could reflect local iron metabolism in the prostate to a certain extent. In conclusion, for the first time, we identified the differences in iron metabolism in patients with PI-RADS 3. Although they had similar imaging findings, there were significant differences in iron metabolism in patients. The findings represent a considerable difference in iron metabolism that can be assessed during the diagnosis of PCa. The results showed that the levels of three indexes of iron metabolism were positively correlated with ISUP, suggesting that under similar imaging conditions, the degree of active iron metabolism in the tumor represents the prognosis of patients with PCa to some extent. It is interesting to note that there is also a significant negative correlation between the T2* value and the levels of three indicators of iron metabolism in patients with PCa, of which Fe concentration is the most significant, which means that the T2* value is similar to the prediction of liver iron deposition in the field of urology.

We assessed iron metabolism in prostate disorders using the T2* mapping sequence in mpMRI. We found that the T2* value was lower in the PCa group than in BPH group (p<0.001). In the PCa group, the T2* value was negatively correlated with ISUP grade, and patients with ISUP>2 tended to have a lower T2* value. Compared to the traditional ADC value, the TPSA and T2* values had better performance in distinguishing PCa and BPH and in distinguishing ISUP ≤ 2 and ISUP>2 (AUC=0.865 and 0.867, respectively). As a traditional PCa diagnostic index, TPSA still has good performance in diagnosing PCa (AUC=0.746), but there was no significant difference within the PCa group (p=0.08). The ADC value is used as a quantitative indicator; however, in identifying PCa, the diagnostic performance was not as good as TPSA or the T2* value (AUC=0.647), but it was able to achieve greater diagnostic performance within the PCa group (AUC=0.729), which is consistent with previous papers showing that the ADC value can be used to predict PCa staging, grading and prognosis (29, 30).

To verify whether the T2* value can represent iron metabolism, we added three iron metabolism-related indicators, hepcidin, ferritin, and Fe, to our study and found that the levels were significantly different between PCa and BPH (p<0.01). Notably, within the PCa group, the three metrics were still significantly different between the ISUP groups (p=0.002, 0.012, 0.001, respectively). In further ROC curve analysis, Ferritin (AUC=0.704), Hepcidin (AUC=0.667) and Fe (AUC=0.748) showed good performance in the diagnosis of prostate cancer. When considering the risk stratification of prostate cancer, the diagnostic efficiency of the Ferritin (AUC=0.743), Hepcidin (AUC=0.7) and Fe (AUC=0.771) is further improved, suggesting that even in patients with prostate cancer, different progression often has different iron metabolism. In the PCa group, the three indexes were all negatively correlated with the T2* value, indicating that the T2* mapping sequence could reflect iron metabolism in PCa to a certain extent and reflect the progression of the disease.

Through this study, we hope to drive the adoption of radiomics and metabonomics in the management of current PI-RADS3 patients. From a radiological standpoint, it might increase the overall diagnostic efficiency; on the other hand, it might allow us to rule out unnecessary biopsies from a clinical perspective, avoiding the risk of possible complications in selected patients.

Inevitably, this article has some limitations. First, the sample size was relatively small, and it is necessary to conduct a multicenter, prospective, large-scale study to confirm the current conclusions. Second, the article lacks prognostic follow-up data, and whether the T2* value can be used as a prognostic indicator remains to be explored. Finally, although the accuracy of sampling was adequate, there are still uncontrollable corresponding errors, and it is necessary to wait for better sampling methods.

## 5 Conclusion

In conclusion, the validation of tissue extraction and metabolic analysis based on T2* Mapping sequence could provide a practical basis for non-invasive preoperative evaluation of patients with prostate malignancies using this technology, and could provide the possibility to discover potential iron metabolism-related therapeutic targets in the future.

## Data availability statement

The raw data supporting the conclusions of this article will be made available by the authors, without undue reservation.

## Ethics statement

The studies involving human participants were reviewed and approved by the Medical Ethics Committee of the First Affiliated Hospital of Soochow University (Suzhou, China; 2021; No. 133). The patients/participants provided their written informed consent to participate in this study.

## Author contributions

WD analyzed data and wrote the manuscript. YH and XZ developed the project. XW, WZ and GL edited the manuscript. GZL and YL performed MRI examinations. All authors contributed to the article and approved the submitted version.
